# Aerobic methane oxidation under copper scarcity in a stratified lake

**DOI:** 10.1038/s41598-019-40642-2

**Published:** 2019-03-18

**Authors:** Carole Guggenheim, Andreas Brand, Helmut Bürgmann, Laura Sigg, Bernhard Wehrli

**Affiliations:** 1 Institute of Biogeochemistry and Pollutant Dynamics, ETH Zurich, 8092 Zurich, Switzerland; 2 Eawag, Swiss Federal Institute of Aquatic Science and Technology, 6047 Kastanienbaum, Switzerland; 3 Eawag, Swiss Federal Institute of Aquatic Science and Technology, 8600 Dübendorf, Switzerland

## Abstract

Aerobic methane-oxidizing bacteria (MOB) substantially reduce methane fluxes from freshwater sediments to the atmosphere. Their metalloenzyme methane monooxygenase (MMO) catalyses the first oxidation step converting methane to methanol. Its most prevalent form is the copper-dependent particulate pMMO, however, some MOB are also able to express the iron-containing, soluble sMMO under conditions of copper scarcity. So far, the link between copper availability in different forms and biological methane consumption in freshwater systems is poorly understood. Here, we present high-resolution profiles of MOB abundance and pMMO and sMMO functional genes in relation to copper, methane and oxygen profiles across the oxic-anoxic boundary of a stratified lake. We show that even at low nanomolar copper concentrations, MOB species containing the gene for pMMO expression are present at high abundance. The findings highlight the importance of copper as a micronutrient for MOB species and the potential usage of copper acquisition strategies, even under conditions of abundant iron, and shed light on the spatial distribution of these microorganisms.

## Introduction

Aerobic methane-oxidizing bacteria (MOB) are phylogenetically diverse and mainly group among the *Alpha*- and *Gammaproteobacteria* (α-MOB and γ-MOB) and the *Verrucomicrobia*. They efficiently mitigate the emission of methane (CH_4_) generated in freshwater systems while utilizing CH_4_ as their sole carbon and energy source^1^. The enzyme methane monooxygenase (MMO) plays a key role for this process by catalysing the first oxidation step, the conversion of CH_4_ to methanol under ambient conditions. Two forms have been described, the soluble MMO (sMMO) and the membrane-bound, particulate MMO (pMMO)^2^. Whereas most known MOB express pMMO, sMMO production has been characterized in only a few organisms^3^. The conserved gene segments *mmoX* and *pmoA* encode subunits of sMMO and pMMO, respectively, and serve as biological markers to track MOB in environmental samples^3^. sMMO has a well-characterized di-iron catalytic centre^2^, but the atomic structure of the pMMO active site is still a matter of debate. Several competing models with different metals and different numbers of metal atoms at the active site of pMMO have been proposed^4–6^. However, it is generally agreed that pMMO is a copper-dependent enzyme. Copper (Cu) has a regulatory effect on MOB activity, especially on the biosynthesis of the pMMO and sMMO enzymes and switching between these in cells able to express both^7^. According to experiments with axenic cultures, sMMO expression occurs under low Cu to biomass levels (<1 µM), whereas pMMO is predominant at concentrations above 5 µM^2,8^. Nevertheless, in cells grown under sMMO expressing conditions, low but detectable levels of pMMO transcription have been measured^9,10^. Well-defined incubation experiments with environmental samples documented the influence of Cu on the MOB community structure and abundance and composition of functional gene transcripts^11,12^. Cu addition stimulated *pmoA* gene transcription and promoted growth of MOB, which mostly lacked *mmoX*. Some MOB utilize special mechanisms to regulate their Cu homeostasis, also in response to Cu toxicity at high levels. The chalkophore methanobactin, the extracellular component of a Cu acquisition system, binds Cu with high affinity and specificity and is able to increase the bioavailable Cu fraction by dissolving Cu from soluble, mineral, and humic sources, but open questions about its role still remain^13,14^. It has recently been proposed that methanobactin works in concert with a protein called MmoD to modulate the Cu-switch of sMMO and pMMO^15^, however, other proteins are also involved in the Cu or Cu-methanobactin uptake and/or the Cu-switch in MOB^16–20^.

The hypothesis that Cu acts as a controlling variable for the distribution of MOB with different enzymatic pathways has not yet been tested under *in-situ* conditions in aquatic systems. Many studies have assessed the role of growth substrates and physical parameters on MOB activity^7,21,22^, but it remains unclear if bioavailable Cu limits the distribution of MOB and affects the dominant enzymatic pathways of CH_4_ oxidation in natural systems^7,14,23^. We make a first attempt to reduce this uncertainty by combining trace metal speciation measurements with marker-gene based analysis of MOB in the water column of a stratified lake. We explore the hypothetical link between the depth distribution patterns of different Cu fractions and the abundance of MOB and their functional genes.

## Results

### Copper and methane oxidation in the water column of Rotsee

**Figure 1:**
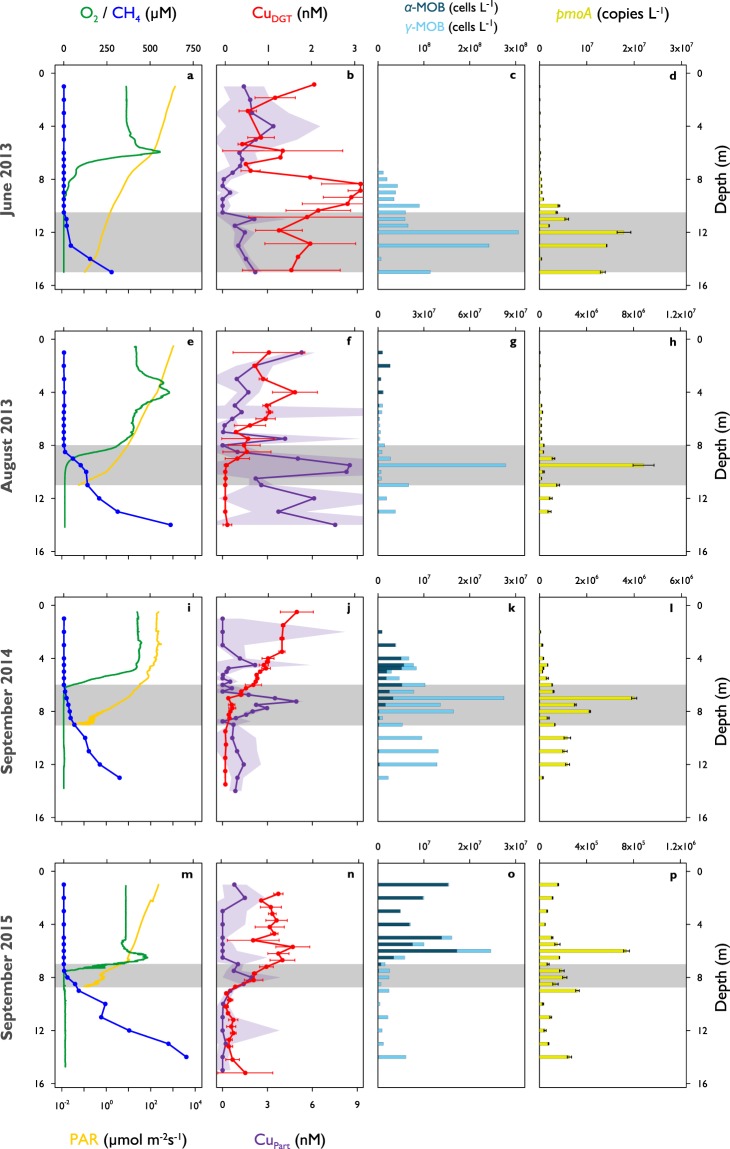
Depth profiles of biogeochemical parameters in Rotsee. (**a**–**d**) June 2013. (**e**–**h**) August 2013. (**i**–**l**) September 2014. (**m**–**p**) September 2015. Grey shaded areas denote the depth range of potential methane oxidation (availability of methane above epilimnetic background concentrations and availability of oxygen or light). (**a**,**e**,**i**,**m**) Oxygen (O_2_, normal optode, green) and methane (CH_4_, blue) concentrations, photosynthetically active radiation (PAR, deep yellow, logarithmic scale). (**b**,**f**,**j**,**n**) Bioavailable copper (Cu_DGT_, red) and particulate copper concentrations (Cu_Part_, purple). Red error bars and purple shaded areas represent standard deviations (n = 3 or n = 4, error propagation for Cu_Part_). (**c**,**g**,**k**,**o**) Absolute abundances of methane-oxidizing bacteria (MOB) separated into α-MOB (deep blue) and γ-MOB (light blue). (**d**,**h**,**l**,**p**) Absolute quantity of *pmoA* gene copy numbers (yellow). Error bars indicate standard deviations from triplicate qPCR amplification of one sample. Note the different x-axes for (**c**,**g**,**k**,**o**) and (**d**,**h**,**l**,**p**).

In order to improve our understanding of CH_4_ and Cu dynamics *in-situ*, we studied seasonally stratified Rotsee, a small freshwater lake (1 km^2^) with pronounced sedimentary CH_4_ production and aerobic CH_4_ oxidation in its water column^24^. We conducted four field campaigns at the early stage (June 2013), peak (August 2013), and end of stratification before the lake overturns (September 2014 and September 2015). CH_4_ concentrations were highest close to its production site in the sediment (270–710 µM) and steadily decreased towards the oxycline where CH_4_ was predominantly consumed (Fig. 1a,e,i,m). The low residual CH_4_ concentrations (0.12–1.07 µM) detected in the oxic epilimnion during all campaigns indicated that CH_4_ removal within the water column of Rotsee was highly efficient. Nevertheless, surface water was still oversaturated in CH_4_ relative to the atmosphere and thus Rotsee was a source of CH_4_. For all campaigns, we found that oxygen (O_2_) concentrations dropped from ~400 µM at the surface to below detection limit (<20 nM) at the oxycline, which varied in depth between 6 m to 9 m across sampling dates (Fig. 1a,e,i,m). Measurements of photosynthetically active radiation (PAR) showed light penetrating throughout the oxycline and into the anoxic zones (Fig. 1a,e,i,m). These findings are consistent with the work of Brand *et al*.^25^, who reported oxygenic primary production in anoxic zones, and Oswald *et al*.^26^, who showed MOB activity consuming O_2_ in these same layers. This indicates that aerobic CH_4_ oxidation might be coupled to oxygenic photosynthesis in the macroscopically anoxic hypolimnion of Rotsee.

We focused our field sampling on depths with the greatest potential for active CH_4_ oxidation: from where CH_4_ first begins to accumulate in the water column, down to where PAR falls below detection limit (grey shaded areas in all profiles, Table 1). Previous work also showed that within these zones, CH_4_ isotopic signatures became substantially heavier, indicative of intense biological CH_4_ oxidation (August 2013, September 2014)^26,27^. Further, isotopic CH_4_ values also showed slight increases in the anoxic, dark hypolimnion of Rotsee, which implies that some CH_4_ is already oxidized at these depths. O_2_ was completely consumed in the upper part of the specified zones, except for June 2013 where O_2_ and CH_4_ gradients did not show any overlap and thus no O_2_ was measured in the potential CH_4_ oxidation zone.

**Table 1:** Methane-oxidizing bacterial cell numbers, copper pools, copper fluxes, and resulting accumulation times in Rotsee’s methane oxidation zones.

Parameter		June 2013 (10.5–15 m)	August 2013 (8–11 m)	September 2014 (6–9 m)	September 2015 (7–8.7 m)
Integrated relative MOB	(%)	6.0	1.4	1.7	0.9
Integrated absolute MOB	(cells m^−2^)	3.8 × 10^11^	5.5 × 10^10^	6.6 × 10^10^	2.8 × 10^9^
Cu_Part_ pool contributed by MOB	(nmol m^−2^)	14	2	1	0.1
Cu_Part_ pool in Rotsee	(nmol m^−2^)	4100	13′100	5200	2400
Cu_DGT_ flux	(nmol m^−2^ d^−1^)	19	30	20	40
Cu_Diss_ flux	(nmol m^−2^ d^−1^)	195	336	828	394
Accumulation time (Cu_DGT_)	(d)	212	433	254	60
Accumulation time (Cu_Diss_)	(d)	21	39	6	6

**Figure 2:**
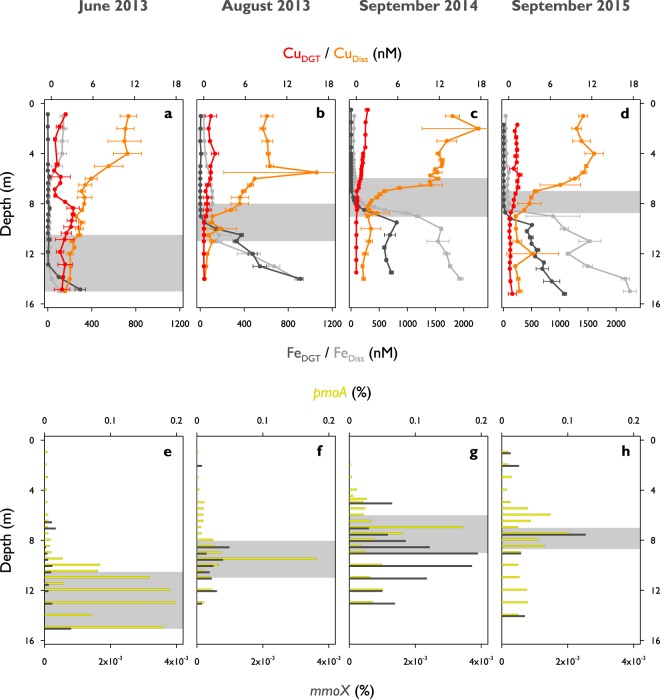
Bioavailable and dissolved metal concentrations and relative methane monooxygenase functional gene abundances in Rotsee. (**a**,**e**) June 2013. (**b**,**f**) August 2013. (**c**,**g**) September 2014. (**d**,**h**) September 2015. The grey boxes show the depth range of potential methane oxidation (see Fig. 1). (**a**–**d**) Depth profiles of bioavailable and dissolved copper (Cu_DGT_ in red, Cu_Diss_ in orange) and iron (Fe_DGT_ in dark grey, Fe_Diss_ in light grey) concentrations. Cu_DGT_ concentrations are identical to Fig. 1. Error bars represent standard deviations (n = 3 or n = 4). (**e**–**h**) Relative abundances of *pmoA* (considering two *pmoA* genes per MOB, yellow) and *mmoX* (dark grey).

Numbers in parentheses denote the depth range of the defined methane oxidation zones (grey zones in Figs 1 and 2). Absolute and relative methane-oxidizing bacterial (MOB) cell numbers as well as particulate copper (Cu_Part_) concentrations were integrated over the methane oxidation zones. Cu_Part_ stemming from MOB was calculated using MOB cell counts and the MOB copper content (4 × 10^−20^ mol Cu cell^−1^) developed from literature values. Accumulation times were estimated by applying bioavailable copper (Cu_DGT_) or dissolved copper (Cu_Diss_) fluxes calculated into the zones on the measured Cu_Part_ pool in Rotsee.

To assess the biogeochemical role of Cu on CH_4_ oxidation, several metal fractions were quantified throughout the water column. We determined bioavailable metals by deploying diffusive gradients in thin-film (DGT) samplers at 0.25–1 m resolution. The DGT technique is based on the diffusion characteristics of different metal chemical species^28^. The samplers allow low molecular weight compounds such as simple inorganic and labile organic complexes as well as free metal ions to diffuse across a diffusive layer to be sorbed at an ion exchange layer. This accumulation process mimics uptake of dissolved metals by organisms. Previous studies showed that the DGT measurement is a good indicator for *in-situ* metal bioavailability^29^. However, the results might underestimate bioavailable Cu as DGT provides a time-integrated concentration and Cu may be bound to large complexes with low diffusion coefficients. Some MOB possess specific uptake pathways such as the ligand methanobactin^13,14^, which may increase the bioavailable Cu fraction.

Maximum bioavailable Cu (Cu_DGT_) concentrations were usually found within the oxic zone of Rotsee (1.6–3.1 nM, Fig. 1b,f,j,n, Supplementary Fig. S1, Supplementary Table S1, Kruskal-Wallis: p < 0.001). The concentrations were about a factor of ten lower than dissolved Cu (Cu_Diss_), which reached concentrations in the low nM range (11.1–17.7 nM, Fig. 2a–d, Supplementary Fig. S1). Cu_DGT_ is expected to be highest where Cu_Diss_ is high. This is typically in the surface layers where Cu originating from river water or surface runoff enters the lake. In addition, concentrations in the epilimnion can be increased due to degradation of organic matter and the release of organically bound Cu. Differences between Cu_DGT_ and Cu_Diss_ might arise due to the bonding of Cu with strong organic complexes, which can pass the 0.45 µm pore size filter and diffuse through the diffusive layer, but cannot be captured by the resin inside the DGT sampling unit^29,30^. Dissolved organic carbon (DOC) profiles were quite homogeneous throughout the water column and do not elucidate any Cu-binding capacity therein (Supplementary Fig. S2). Both Cu fractions clearly decreased from the epi- to the hypolimnion and profiles followed typical patterns previously observed at lower resolutions in other subalpine lakes in Switzerland^30,31^. MOB need to acquire Cu to build and activate pMMO. They can either directly incorporate bioavailable Cu (Cu_DGT_) or enlarge this fraction by the use of different Cu uptake mechanisms^13,16,18^. These auxiliary peptides collect Cu and bind it with high affinity, hence, they are not included in the Cu_DGT_ fraction, and only measured as Cu_Diss_. We monitored strong Cu_DGT_ and Cu_Diss_ gradients towards the CH_4_ oxidation zones where concentrations were depleted (Figs 1b,f,j,n and 2a–d). Calculated particulate Cu concentrations (Cu_Part_ = Cu_Tot_ − Cu_Diss_) showed maximum values within the CH_4_ oxidation zones (1.9–8.5 nM, Fig. 1b,f,j,n, Supplementary Fig. S1, Supplementary Table S1, Kruskal-Wallis: p < 0.05), which matches well with the decreases in Cu_DGT_ and Cu_Diss_. Cu_Part_ showed some additional local peaks within the other zones (Fig. 1b,f,j,n, Supplementary Fig. S1).

### MOB zonation and functional gene distributions

To link physico-chemical conditions with microbial CH_4_ consumption dynamics, we quantified bacterial 16S rRNA and functional gene (*pmoA* and *mmoX*) distributions in Rotsee down to 0.5 m resolution. 16S rRNA sequencing yielded a total number of 3679 Operational Taxonomic Units (OTUs). In-depth analysis of the OTU data set revealed a total of 15 OTUs belonging to γ-MOB and one α-MOB OTU (Supplementary Table S2). *Verrucomicrobia* were also detected and were represented by 5 OTUs. 3 of the 5 OTUs were assigned to potentially methanotrophic clades (p ≤ 0.85) while the remaining two belonged to a family known not to contain any MMO (LD19), which hence probably lack the ability of oxidizing CH_4_^32^. As the applied primer pair for *pmoA* detection did not cover verrucomicrobial *pmoA* sequences, and genomic investigations of freshwater *Verrucomicrobia* implicated them as (poly)saccharide degraders^33^, the verrucomicrobial OTUs were not included in the further analysis of the data.

MOB diversity was highest in September 2014 and 2015, when richness peaked at 15 OTUs, whereas in June and August 2013, the MOB community consisted of 10 and 11 OTUs, respectively (Supplementary Table S2). The single α-MOB OTU was found in all campaigns, but with variable abundance. This OTU appeared primarily in the epilimnion and was therefore unlikely to participate in the dominant CH_4_ oxidation process in Rotsee (Fig. 1c,g,k,o). The MOB community was dominated by γ-MOB, which is in agreement with previous studies on Rotsee and other (sub)alpine lakes, suggesting that γ-MOB play an important role in freshwater CH_4_ cycling^24,26,27,34,35^. Although most so far characterized γ-MOB are obligate aerobes, we could detect them in suboxic and anoxic layers throughout the campaigns. We found maximum absolute MOB cell numbers (10^6^–10^7^ cells L^−1^) directly below the oxycline in August 2013 and September 2014 (Fig. 1g,k), which supports our assumption that MOB abundance peaks at depths where physico-chemical profiles indicate active aerobic CH_4_ oxidation. Within the CH_4_ oxidation zones the proportion of MOB reached 1.2–3.4% of the total bacterial abundance.

In June 2013, most MOB (10^8^ cells L^−1^) were found in deeper layers where neither O_2_ nor oxygenic phototrophs were detected (Fig. 1c)^25^. Several mechanisms may allow MOB to persist under these conditions. It is possible that some MOB from the oxic period during the winter mixing remained in the deeper parts of the lake or that they are settling from upper, oxygenated water layers. MOB could potentially be inactive under anoxic conditions, as it is known that MOB can enter a state of anaerobic dormancy for extended periods of O_2_ starvation^36^. However, the methodology applied in this study cannot determine the activity of the cells. Some MOB species in the dark, anoxic layers of Rotsee may be able to survive O_2_-limiting conditions while using fermentation as their main metabolic strategy^37^. MOB may live mixotrophically or use other reduced carbon compounds as alternative energy sources (facultative MOB)^38^. Or, they could be involved in anaerobic oxidation of CH_4_ (AOM) and use O_2_ or other electron acceptors provided by different reaction pathways^39,40^. Anaerobic methanotrophic archaea could also play a role in oxidizing CH_4_ at depths depleted in O_2_^41^, however, in this study DNA was only screened for bacteria. MOB distribution in September 2015 showed an abundance maximum above the oxycline with a majority of α-MOB (10^7^ cells L^−1^, Fig. 1o). There is evidence that littoral sediments can act as additional source of MOB in the water column^42^. Alternatively, MOB may accumulate due to *in-situ* CH_4_ production in the oxygenated epilimnion, a phenomenon that has been frequently observed^43^.

The potential involvement of particulate and soluble MMO was identified by the quantitative detection of *pmoA* and *mmoX*, respectively. As most MOB contain *pmoA*, these results can independently confirm MOB abundance evaluated from 16S rRNA sequencing data. Real time quantitative polymerase chain reaction (qPCR) yielded *pmoA* copy numbers between 10^4^–10^7^ copies L^−1^ (Fig. 1d,h,l,p). The depth-distribution of *pmoA* counts correlated well with MOB concentrations from 16S rRNA based community analysis, resulting in R^2^-values of 0.89 (June 2013), 0.98 (August 2013), 0.87 (September 2014) and 0.40 (September 2015). However, absolute numbers suggested by *pmoA* analysis were on average a factor of ~30 lower than those derived from 16S rRNA gene analysis, possibly a result of methodical biases. Correspondingly, the calculated proportion of cells containing copies of *pmoA* was between 0.002–0.2% (Fig. 2e–h).

Relative abundances of *mmoX* were on average 160 times lower compared to *pmoA* and remained below the limit of quantification for some samples (Fig. 2e–h). This indicates that we never observed a population with *mmoX* becoming a numerically important part of the MOB community. It has been reported that cells expressing pMMO have a higher growth yield and greater affinity forCH_4_ than cells relying on the sMMO mechanism^44,45^, which suggests that pMMO is the more efficient system. *mmoX* was usually found in the hypolimnion of Rotsee. The highest relative abundance of *mmoX* was observed in September 2014 (Fig. 2g), and this was the only time a clear *mmoX* peak was observed ~2 m below the *pmoA* maximum, towards the lower end of the CH_4_ oxidation zone. This coincided with minimum Cu and highest iron availability (Fe_DGT_ and Fe_Diss_; Fig. 2a–d) and could support the notion that MOB populations with this gene grow under Cu-limited conditions at this specific time point. However, correlations between MOB and *mmoX* distributions were rather low over all campaigns (R^2^ = 0.00–0.54). We propose that sMMO-mediated CH_4_ oxidation is of minor importance in Rotsee at the investigated sampling dates, although additional studies are needed to confirm this.

Having a closer look on the specific CH_4_ oxidation zones of August 2013 and September 2014, the concentrations of *pmoA* genes rapidly increased at the upper boundary of the zones in which they showed highest abundances (Fig. 1h,l). Their concentrations increased in parallel with Cu_Part_ (Fig. 1f,j). In September 2014 the correlation between *pmoA* and Cu_Part_ within the CH_4_ oxidation zone was high (R^2^ = 0.85), however, R^2^-values for the other three campaigns were much lower (June 2013: 0.22, August 2013: 0.30, September 2015: 0.06) indicating that MOB did not constitute the main component of the Cu_Part_ concentrations.

### Biogeochemical fluxes and competition for copper in the methane oxidation zone

We computed fluxes of various solutes into the CH_4_ oxidation zones from measured concentration gradients and a site-specific coefficient for turbulent diffusion (Supplementary Table S3). Dissolved O_2_ concentrations in the epilimnion of Rotsee gradually decreased towards the oxycline (Fig. 1a,e,i,m). O_2_ downward fluxes showed the largest change, from lowest values in June 2013 when stratification was still in its early stage (3.4 mmol m^−2^ d^−1^), to about tenfold higher ones in the other three campaigns (27.6–42.9 mmol m^−2^ d^−1^). These O_2_ flux estimates do not account for potential oxygenic photosynthesis within the seemingly anoxic CH_4_ oxidation zone^25^. Theoretical CH_4_ fluxes out of the sediment were about half to one order of magnitude lower than for O_2_ (2.7–13.3 mmol m^−2^ d^−1^), except for June 2013 (10.2 mmol m^−2^ d^−1^), which indicates O_2_ is being consumed by additional processes, like mineralization and oxidation of other reduced substances. Dissolved and bioavailable Cu fluxes from the lake’s surface down to where they are depleted were in the nmol m^−2^ d^−1^ range, with values for Cu_Diss_ being about ten times higher than for Cu_DGT_ (Table 1). The Cu_Part_ pool integrated over the CH_4_ oxidation zones ranged from 2.4 to 13.1 µmol m^−2^ (Table 1). As Rotsee typically starts to re-stratify in April^25^, it has been stratified for approximately 60 d in June 2013, 120 d in August 2013 and 160 d in September 2014 and 2015. We calculated a rough estimate of the Cu_Part_ build-up times based on Cu_Diss_ fluxes (6–39 d; Table 1) and the results indicate that Cu_Part_ build-up would have required much less time than the stratification period. Thus, organisms or particle formation processes either cannot access the Cu_Diss_ fraction, or else Cu_Part_ settling rates are extremely high. In contrast, accumulation times estimated for Cu_DGT_ fluxes (212–433 d, Table 1) were much longer than the time elapsed since the onset of stratification (with exception of September 2015 with 60 d). This indicates that either organisms in the CH_4_ oxidation zones established mechanisms to mobilize Cu from other not directly available sources, or that the bioavailable Cu values measured by the DGT method were underestimating the true bioavailable concentrations.

In order to assess whether MOB are an important contributor to Cu_Part_ in Rotsee’s CH_4_ oxidation zones, we calculated an extended Redfield stoichiometry (Cu:C) for a single MOB cell. The Redfield ratio stands for the specific elemental composition (C:N:P) of a cell reflecting the conditions under which it grows^46^. Assuming an average MOB spherical cell diameter of 2 μm, the biovolume of a single cell (μm^3^ cell^−1^) can be converted into biomass (mol carbon cell^−1^) by applying a carbon (C) conversion factor of 6.4 fmol C μm^−3^ ^26^. This results in a C content of 0.03 pmol C cell^−1^. Considering the studies focusing on the type and number of Cu ions in pMMO’s metal centre, we choose a range of 2–15 Cu ions per pMMO^4^. Accepting the estimate of Nihous by which cell membrane walls are able to bind between 1000–4000 pMMO enzymes^47^, a single MOB organism contains between 2000–60′000 Cu atoms in pMMO, which amounts to a mean of 4 × 10^−20^ mol Cu cell^−1^. Putting this value in relation to the calculated C content, we obtained an averaged proportion for Cu:C of 10^−6^. This ratio is more likely to be an under- than an overestimate since MOB cells contain Cu acquisition and storage proteins and use Cu also for other enzymes besides pMMO^13,16,17,19,20,23^. Nevertheless, based on these cellular Cu contents we calculated a contribution of MOB to Cu_Part_ that underestimates the actually measured *in-situ* Cu_Part_ concentrations by a factor of 10^2^–10^4^ (Table 1). Even though MOB have an overall stronger Cu demand than other organisms (~10 times)^7,14,23^, and we saw agreement in the distributions of MOB, *pmoA* and Cu_Part_ at expected CH_4_ oxidation zones, we conclude that they contributed only a small part of the measured Cu_Part_ in Rotsee.

**Figure 3:**
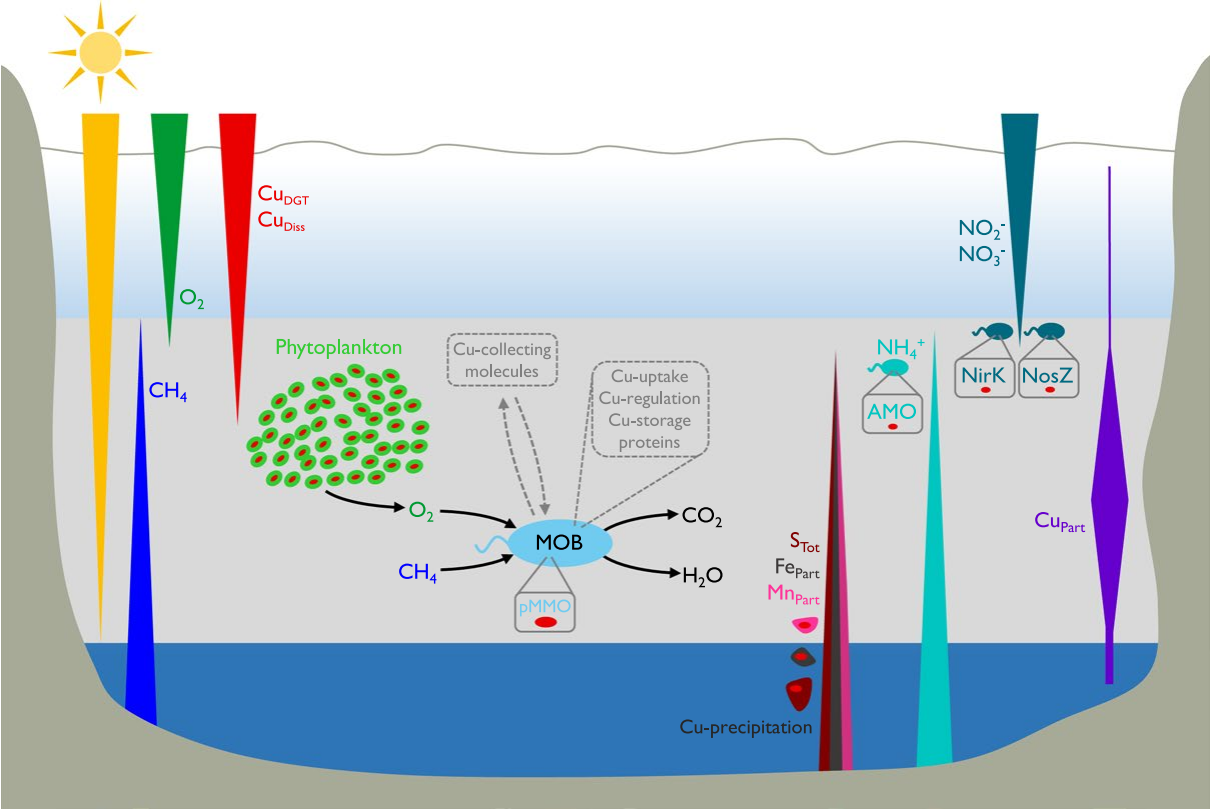
Long triangles depict concentration gradients of different parameters across the water column. Bioavailable and dissolved copper (Cu_DGT_, Cu_Diss_) diffuse into the methane (CH_4_) oxidation zone (grey bar) and are depleted by different processes, which contribute to elevated particulate copper (Cu_Part_). Methane-oxidizing bacteria (MOB) are highly abundant in the CH_4_ oxidation zone and need to compete for Cu, the fundamental micronutrient for their major enzyme particulate methane monooxygenase (pMMO). Phytoplankton is the main constituent of Cu_Part_ and the main competitor for Cu. However, its presence is at the same time crucial for MOB as it provides O_2_ under low light conditions in anoxic depths, which is used by MOB. Cu can also be incorporated into other bacteria, for example ammonia-oxidizing bacteria and bacteria involved in denitrification. They use Cu for their central enzymes ammonia monooxygenase (AMO) and nitrite reductase (NirK)/nitrous oxide reductase (Nos). Further, Cu can be captured by rising sulphide (S_Tot_) and precipitate to the sediment as CuS, or Cu can be scavenged by precipitating iron and manganese oxides (Fe_Part_, Mn_Part_). Therefore, MOB probably make use of Cu scavenging mechanisms, i.e. the release of Cu-collecting molecules (methanobactin or MopE*) and the involvement of Cu-uptake (CorA), Cu-regulation (CopCD) and Cu-storage proteins (Csp).

Cu holds an important role in the photosynthetic and respiratory electron transport in phytoplankton^48^. Extended Redfield ratios of marine and freshwater phytoplankton centre around a total Cu content of 10^−16^–10^−18^ mol cell^−1^ and a Cu:C ratio of ~10^−6^ ^49–51^. This Cu:C ratio is similar to the theoretical stoichiometric ratio of a MOB cell, but phytoplankton, being significantly larger than MOB, contains much more Cu per cell (factor 10^2^–10^4^). With the exception of June 2013, Cu_Part_ concentrations showed distinct peaks within the defined CH_4_ oxidation zones, which coincided well with chlorophyll a (Chl-a) maxima and turbidity (Turb) measurements (Supplementary Fig. S3), both suitable proxies for phytoplankton abundance. As primary producers were present in similar numbers as MOB in Rotsee (~10^7^ cells L^−1^)^25^, we suggest that the difference in Cu content between MOB and phytoplankton explains most of the observed discrepancy between measured Cu_Part_ and the calculated Cu_Part_ contribution of MOB within the defined zones (Fig. 3). In June 2013, lower Cu_DGT_ concentrations in the epilimnion might illustrate the uptake by phytoplankton at ~6.5 m (Fig. 1b, Supplementary Fig. S3). This corresponded to the slight decrease in Cu_Diss_ at similar depths (Fig. 2a). However, a second distinct Chl-a increase at deeper depths was missing, indicating an overall weaker Cu-consumption by primary producers, which resulted in elevated Cu_DGT_ concentrations.

These findings lead to the conclusion that MOB are strongly enriched within highest CH_4_ oxidation zones despite low bioavailable Cu supply and co-occurrence of large numbers of oxygenic primary producers, which possibly create a setting of Cu competition. MOB cannot escape the Cu scarcity as in the study system they also rely on oxygenic primary production for O_2_ supply (Fig. 3)^25,26^. The low Cu availability may at first glance appear as an impediment to the growth of MOB. Nevertheless, it has been suggested that the competitiveness of MOB in terms of Cu acquisition may in fact favour their growth^52^. Cu is also an essential element for many other bacteria and organisms^53^. Enzymes involved in the denitrification process, in particular the nitrite reductase (NirK) and the nitrous oxide reductase (Nos), are Cu-rich proteins^23^. Nitrite (NO_2_^-^) and nitrate (NO_3_^-^) concentrations in Rotsee were low (max. 30 µM) and it seems that the organisms containing these proteins potentially only make a small contribution to Cu_Part_ (Supplementary Fig. S4). In addition, some MOB species also contain the gene for the nitrite reduction process (NirK)^27^. The closely related pMMO homologue, ammonia monooxygenase (AMO), belongs to the Cu-containing membrane-bound monooxygenase (CuMMO) superfamily and catalyses the initial ammonia (NH_3_) oxidation step^54^. Inferred sites of NH_4_^+^ oxidation or uptake by primary producers in Rotsee only weakly overlapped with Cu_Part_ maxima (Supplementary Fig. S5).

Alternative mechanisms for Cu_Part_ formation might include abiotic reactions, such as iron and manganese oxide scavenging or precipitation with sulphide (Fig. 3)^55^, but we found little evidence that any of these processes are dominant in Rotsee. Profiles of particulate iron (Fe_Part_) and manganese (Mn_Part_) did not appear to be strongly related to the Cu_Part_ profiles (Supplementary Fig. S6). Sequencing data of sulphur oxidizing bacteria (SOB) showed highest relative abundances at the upper hypolimnion where light was still measurable (Supplementary Fig. S7). We assume that total sulphide (S_Tot_) is continuously consumed by SOB during the day during which sampling campaigns were conducted^56^ and thus resulting Cu_Part_ precipitation with S_Tot_ would be minimal.

## Discussion

Our findings have important implications for CH_4_ oxidation in lakes and specifically for Cu-dependent MOB activity. During stratification, MOB in Rotsee were abundant within a zone with CH_4_ supply from the sediment and O_2_ release from oxygenic photosynthesis (Fig. 3). Most of the CH_4_ generated in the sediment was oxidized at this oxic-anoxic boundary. CH_4_ concentrations could have additionally been reduced within the hypolimnion of the lake, however, in this study we did not specifically assess the potential for anaerobic oxidation of CH_4_. Bioavailable and dissolved Cu concentrations were in the nanomolar range and showed strong depletion in the zone of CH_4_ oxidation. Far higher *pmoA* copy numbers, coding for the Cu-containing enzyme, compared to *mmoX* were found in the whole water column at all times. The dominant MOB species therefore had to cope with Cu scarcity and they were not the only competitors. Other organisms, particularly oxygenic phototrophs, possibly competed for Cu in the CH_4_ oxidation zones (Fig. 3). However, MOB could have used several mechanisms to deal with low Cu supply conditions to maintain pMMO production. The chalkophore methanobactin is able to dissolve Cu from soluble, mineral, and humic sources^13,14^. MopE is a membrane-bound Cu binding protein, and its truncated form (MopE*) is secreted into the environment to collect Cu^16^. MopE* shares sequence resemblance to the CorA (copper repressible) protein. CorA is located on the cell surface and it is postulated to be involved in the uptake of Cu into the cell^17^. A copper resistance protein-mediated (CopCD) Cu uptake may play a role for delivering Cu to the cytosol of the cell^19^. Additional insight in how MOB manage their Cu demand has also been provided by the recent discovery of a new family of Cu storage proteins (Csp)^20^. Such mechanisms are likely to be crucial for MOB ecology in low-Cu environments such as Rotsee.

This detailed *in-situ* study of Cu and CH_4_ oxidation reveals that MOB species containing the gene for a potential Cu-dependent enzymatic pathway are abundant despite low bioavailable Cu concentrations and excess of Fe. We provide evidence that other biological processes and abiotic reactions could have a profound impact on the availability of Cu in the water column, which may in turn have consequences for the ecology of CH_4_ oxidation, a critical process in the global carbon cycle. Since Rotsee is representative for mid-latitude, nutrient-rich lakes in terms of topography and chemical cycling, we expect that the observed distribution of Cu species as well as the vertical zonation of MOB and phytoplankton is typical for such lakes. Therefore, the proposed Cu competition and inferred mechanisms of MOB adaptation to Cu scarcity may be common in numerous other lakes.

## Methods

### Study site

Rotsee is a small (1 km^2^), eutrophic subalpine lake in Switzerland with a maximum depth of 16 m. Rotsee exhibits a stable summer stratification from approximately April-November with an oxycline between 6 m and 9 m depth^24,25^. Large amounts of CH_4_ are released from the sediments reaching concentrations up to 1 mM before winter overturn^24^.

### *In-situ* profiling and sampling, chemical analysis

Four sampling campaigns were conducted (June 2013, August 2013, September 2014, September 2015) near the deepest part of Rotsee (47°04.259′N, 8°18.989′E). A custom built device (Profiler for *In-situ* Analysis, PIA)^57^ was used for high resolution profiling and sampling: conductivity, turbidity, depth, temperature and pH were monitored with a CTD multi-parameter probe and Chl-a with an ECO-FL fluorescence probe (Wetlabs). Profiles of dissolved oxygen (O_2_) concentrations were obtained from two needle-type optodes (PSt1 and TOS7, PreSens) with detection limits of 125 nM (normal) and 20 nM (trace), respectively. Photosynthetically active radiation (PAR) was recorded with a spherical quantum sensor (LI-190 SB, LI-Cor). Detection limit of PAR sensing photon flux was 0.1 µmol m^−2^ s^−1^. Water for chemical analysis was taken using an integrated rosette syringe sampler (12 × 60 ml syringes), which could be triggered remotely during profiling. Sampling was carried out across the whole water column with high resolution in the oxycline (0.25–0.5 m) and in one-meter steps otherwise. Aliquots for sulphate (SO_4_^2−^), nitrite (NO_2_^−^), nitrate (NO_3_^−^) and ammonium (NH_4_^+^) were filtered (0.22 μm cellulose acetate syringe filters) and analysed on the same day by ion chromatography (881 Compact IC pro, 882 Compact IC plus, 761 Compact IC, Methrom AG) and flow-injection analysis (SAN++, Skalar, Procon AG). Samples for total sulphide (S_Tot_) detection were immediately fixed with zinc acetate (final concentration: ~1.3%) and determined spectrophotometrically^58^. Equipment for metal sampling and filtering was acid-washed and rinsed with nanopure water before use. Triplicate samples for dissolved (<0.45 μm cellulose acetate syringe filters) and total metals (Cu_Diss_, Cu_Tot_, Fe_Diss_, Fe_Tot_, Mn_Diss_, Mn_Tot_) were acidified on-site to a final concentration of 0.1 M HNO_3_ and analysed with inductively coupled plasma mass spectrometry (ICP-MS, Element2, Thermo). Particulate metal species (Cu_Part_, Fe_Part_, Mn_Part_) were calculated as Cu_Part_ = Cu_Tot_ − Cu_Diss_. Cu_Part_ errors were estimated by standard deviation propagation from dissolved and total metal measurements. Aliquots for dissolved organic carbon (DOC) analysis were filtered (0.22 μm, Millex-GP polyethersulfone syringe filters) into pre-combusted glass vials and acidified with 2 M HCl (final concentration: 20 mM) and measured on a total carbon analyser (TOC-L_CSH/CPH_, Shimadzu) equipped with a non-dispersive infrared detector. Outliers of triplicate samples were removed applying Grubbs’ outlier test. Methane (CH_4_) samples were collected with a Niskin bottle or via pumping with a gas tight tubing (PVC Solaflex, Maagtechnic) attached to PIA. 120 ml serum bottles were filled anoxically, poisoned with NaOH (pH > 12) or Cu(I)Cl, and sealed with butyl-rubber stoppers and aluminium crimps. In the laboratory, a 20 ml nitrogen (N_2_) headspace was inserted. After overnight equilibration at room temperature, CH_4_ was measured by headspace injection using a gas chromatograph (GC, Agilent 6890 N, Agilent Technologies) equipped with a Carboxen 1010 column (30 m × 0.53 mm, Supelco) and a flame ionization detector (detection limit: ~10 nM). Vertical diffusive fluxes of dissolved compounds into the CH_4_ oxidation zones (Supplementary Table S3) were calculated from the chemical concentration gradients determined by linear regression and a vertical turbulent diffusion coefficient of 10^−6^ m^2^ s^−1^ ^24–26^.

### Diffusive Gradients in Thin film gels (DGT)

DGT preparations were performed in a clean room (except for June 2013). All plastic devices, containers and membrane filters were soaked in diluted HNO_3_ for 24 h and rinsed with nanopure water before use. Acrylamide (40%, Sigma Aldrich), agarose cross-linker (2%, DGT Research Ltd.) and nanopure water were used to generate diffusive and resin hydrogels according to Odzak *et al*.^30^. To initiate chemical polymerisation freshly mixed ammonium persulfate (10%, Sigma Aldrich) and TEMED (N,N,N′N′-Tetramethylenediamine, 99%, Sigma Aldrich) were added. The resin hydrogel contained additional ion-exchange resin (Chelex-100, 200–400 mesh, Na^+^ form, Bio-Rad). The gels were hydrated and cleaned with nanopure water and stored in 0.01 M NaNO_3_. Each piston was loaded with a resin gel, a diffusive gel (0.8 mm thickness), a protective filter (0.13 mm thickness, <0.45 μm cellulose nitrate, Sartorius) and a plastic cap (2 cm diameter exposure window; Supplementary Fig. S8). 3–4 DGTs were placed into a plastic stripe and attached to a rope. Deployment time was 2–3 days. Some DGTs were left in the laboratory as controls. After deployment, resin gel layers were eluted in 1 M HNO_3_ for 1–2 days. After dilution (to 0.1 M HNO_3_), bioavailable trace metal concentrations (Cu_DGT_, Fe_DGT_, Mn_DGT_) were analysed via ICP-MS. The accumulated mass of the analytes was calculated following Davison^28^ with published diffusion coefficients (accessed May 2016, http://www.dgtresearch.com/diffusion-coefficients/). The negligible thickness of the diffusive boundary layer was disregarded. Significant outliers were determined based on Grubbs’ outlier test.

### DNA extraction and sequencing of 16S rRNA

Water samples were pre-filtered (<5.0 µm) and subsequently filtered onto 0.2 µm polycarbonate membranes. Filters were packed into plastic bags, immediately frozen in liquid N_2_ and stored at −80 °C until DNA was extracted using a PowerWater^®^ DNA Isolation Kit (MoBio Laboratories). Extracted DNA was quantified using a NanoDrop 1000 Spectrophotometer (Thermo Fisher).

Illumina MiSeq sequencing technology was performed on amplicons obtained with bacterial primers 341f (5′-CCTACGGGNGGCWGCAG-3′) and 805r (5′-GACTACHVGGGTATCTAATCC-3′)^59^. 16S rRNA gene PCRs, library preparation and sequencing were conducted by Microsynth. Sequence data was analysed by the Genomic Diversity Centre (ETH Zurich), which clustered the sequences into operational taxonomic units (OTUs) with a cut-off value of 97% similarity using the Uparse workflow with usearch (v8.1.1812_i86linux64). Taxonomic identity was classified via UTAX based on the GreenGene database (May 2013, http://greengenes.lbl.gov/). Narrowing the data set gave a final alignment of 1 α-MOB, 15 γ-MOB, and 5 potential verrucomicrobial MOB (Supplementary Table S2, Supplementary txt-file “16S rRNA sequences_MOB”). These taxonomic assignments were confirmed against SILVA SSU database (release 123) using RDP classifier (confidence level of 80%) as well as by NCBI megaBLAST against GenBank numbers (https://blast.ncbi.nlm.nih.gov/Blast.cgi). OTUs of sulphur oxidizing bacteria (SOB) were chosen based on the assignments to phylum *Chlorobi* and orders *Chromatiales* and *Legionellales*, and were checked with literature^60^ (Supplementary txt-file “16S rRNA sequences_SOB”).

### Quantification of 16S rRNA, *pmoA* and *mmoX* genes

For analysing 16S rRNA and methane monooxygenase (MMO) functional genes, the limit of detection (LOD) was set as the highest crossing point (Cp-value) determined in PCR or extraction blanks. A sample was considered not detectable if either its Cp-value was ≥ Cp-value LOD or if no clear, or multiple, melting temperature (Tm) peak(s) were detected in comparison to the positive control. The limit of quantification (LOQ) was the concentration of the lowest quantifiable standard dilution with a standard deviation of quadruplicate Cp-values < 0.5. Samples above LOD were described not quantifiable when Cp-values of replicates differed more than 0.5, Cp-values were > LOQ, or when 2 out of 3 replicates were < LOD. However, some sample concentrations were estimated using standard curve extrapolation below LOQ. qPCR efficiency was calculated from the slope of the standard curve (*E* = 10^−1/*slope*^). Product lengths were additionally verified by gel electrophoresis (1.5% agarose gel). All samples were run on a Roche Light Cycler 480 (Roche Diagnostics).

16S rRNA-qPCR reactions were adapted from Takai and Horikoshi^61^ (Supplementary Table S4). 16S rRNA gene copies were used as a proxy for the size of the bacterial community and for translating relative MOB abundances into absolute cell numbers by applying specific amounts of 16S rRNA copies per genome (5.8 for *Gammaproteobacteria*, 2.2 for *Alphaproteobacteria*, 4.2 for other bacteria^62^). Copy numbers of *pmoA* were measured using an adjusted protocol from Henneberger *et al*.^63^ (Supplementary Table S4). qPCR measurements of *mmoX* were performed following the conditions listed in Supplementary Table S4. To determine the calibration curves, plasmids containing amplifiable fragments of each target gene were serially diluted in AE buffer (5 × 10^7^−5 × 10^0^ copies per reaction). All standards were run in quadruplicates, all samples in triplicates. Genomic DNA of several axenic culture strains served as positive and negative controls (Supplementary Table S5). *pmoA* and *mmoX* gene copy numbers were both normalized with bacterial 16S rRNA gene copies^62^, assuming 2 copies per MOB cell for *pmoA*^64^.

### Statistical analysis

The water column of Rotsee was divided into three zones (oxic zone, methane oxidation zone, anoxic zone) to apply statistical testing using the Past3.18 statistic software (http://folk.uio.no/ohammer/past/). Normality of the data was tested by the Shapiro-Wilk test. Differences between the three zones were evaluated using the Kruskal-Wallis test followed by the Mann-Whitney pairwise test if normality was not met. Else, a one-way ANOVA following a Tukey’s HSD test was performed. p-values < 0.05 were considered significant. Tests were conducted for all seasons, and for single seasons each (Supplementary Table S1).

### Nucleotide sequence accession numbers

The gene sequences obtained in this study are publically archived on the ENA/EBI database (accession number PRJEB28460).

## Supplementary information

Supplementary Information

Dataset 1

Dataset 2

## Data Availability

The authors declare that the data supporting the findings of this study are available within the article and its supplementary or from the corresponding author on reasonable request.
