# Supplementary material for: Aerobic methane oxidation under copper scarcity in a stratified lake
Source: Sci Rep. 2019 Mar 18;9:4817. doi: 10.1038/s41598-019-40642-2 (PMC6423226; doi:10.1038/s41598-019-40642-2)
Supplement: Supplementary file 1 — Supplementary Information [file 41598_2019_40642_MOESM1_ESM.pdf]

Supplementary information

Aerobic methane oxidation under copper scarcity in a stratified lake

Carole Guggenheim[1,2 *], Andreas Brand[1,2], Helmut Bürgmann[2], Laura Sigg[3] and Bernhard Wehrli[1,2]

[1]Institute of Biogeochemistry and Pollutant Dynamics, ETH Zurich, 8092 Zurich, Switzerland

[2]Eawag, Swiss Federal Institute of Aquatic Science and Technology, 6047 Kastanienbaum, Switzerland

[3]Eawag, Swiss Federal Institute of Aquatic Science and Technology, 8600 Dübendorf, Switzerland

*e-mail: carole_guggenheim@hotmail.com

Figure S1. Box-Whisker plots of different copper species subdivided into three zones (oxic zone, methane oxidation zone, anoxic zone). a, Cu_{DGT}. **b**, Cu_{Diss}. **c**, Cu_{Part}. Plots combine data from all four field campaigns (June 2013, August 2013, September 2014, September 2015), Boxes denote median and lower/upper quartiles, Whiskers represent 10/90 percentiles. Note the different y-axis ranges.

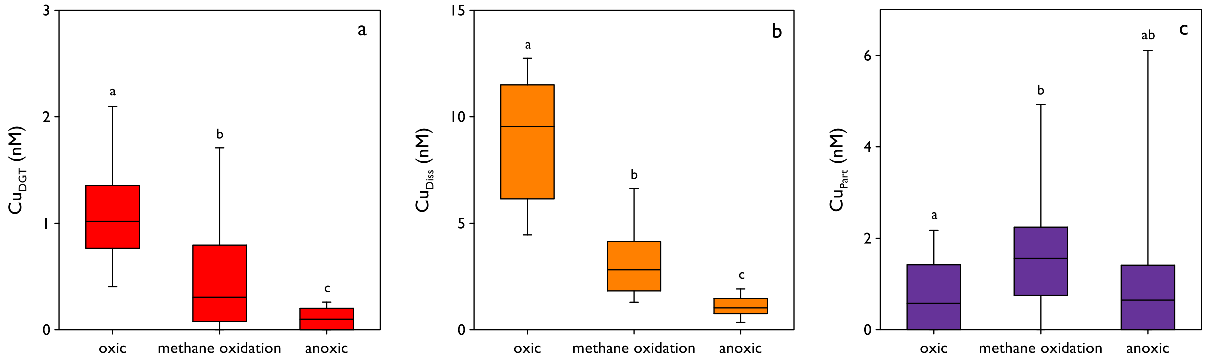

Figure S2. Depth profiles of dissolved organic carbon (DOC) in Rotsee. a, June 2013. **b**, August 2013. **c**, September 2014. **d**, September 2015. Grey areas represent potential methane oxidation zones. Error bars indicate standard deviations (n=3).

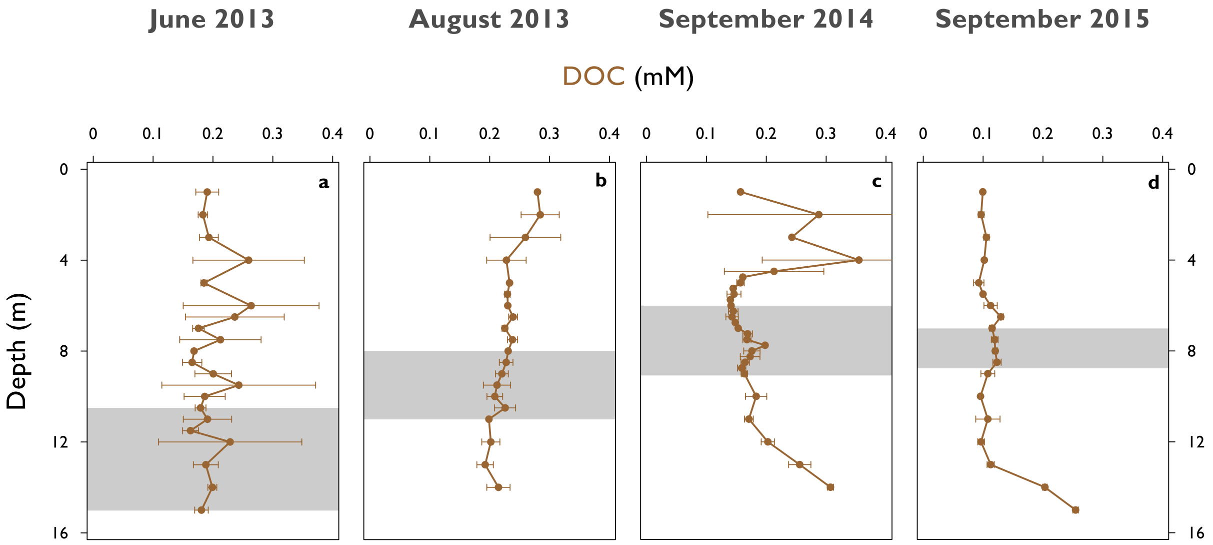

Figure S3. Turbidity, chlorophyll a and particulate copper profiles in Rotsee. a, June 2013. **b**, August 2013. **c**, September 2014. **d**, September 2015. **a**,**b**, Chlorophyll a (Chl-a) measurements are shown in grass green. No Chl-a data are available for September 2014 (**c**) and September 2015 (**d**), respectively. **a-d**, Turbidity (Turb) is drawn in deep green, particulate copper (Cu_{Part}) in purple. Grey bars represent potential methane oxidation zones. Note the different x-axis scale for Cu_{Part} in August 2013 (**b**).

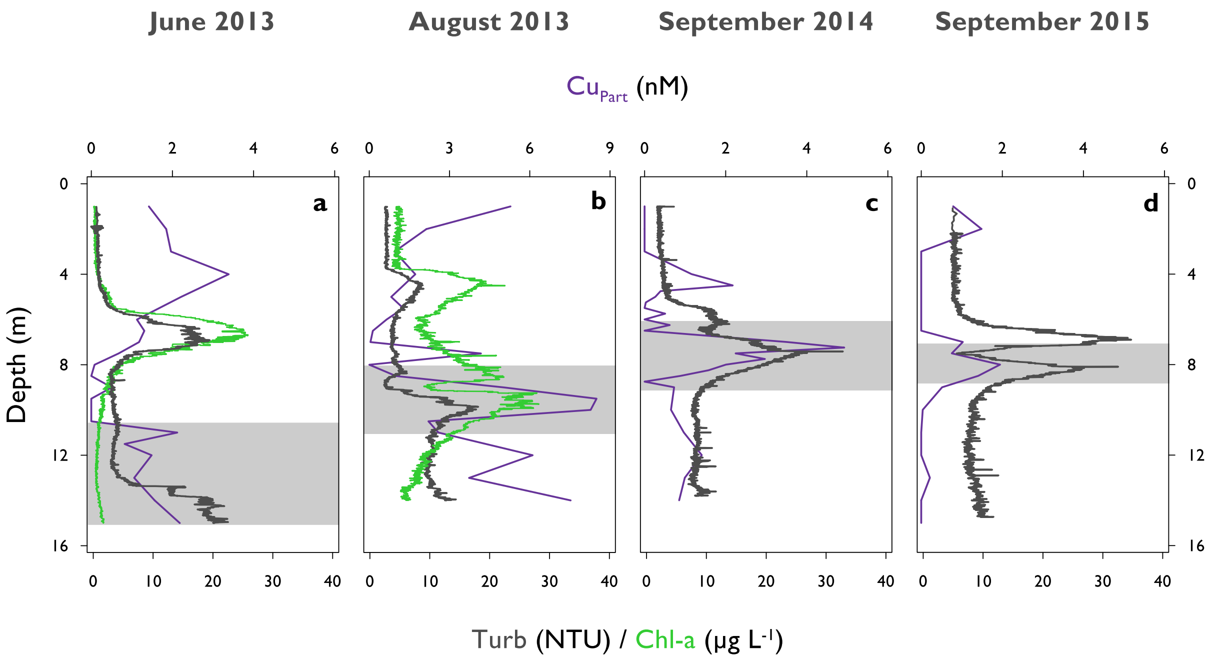

Figure S4. Water column profiles of nitrite, nitrate and particulate copper. **a**, June 2013. **b**, August 2013. **c**, September 2014. **d**, September 2015. Particulate copper (Cu_{Part}) is shown in purple, nitrite (NO_2^-) in dark turquoise, nitrate (NO_3^-) in turquoise. Grey bars represent potential methane oxidation zones. Note the different x-axis scale for Cu_{Part} in August 2013 (**b**).

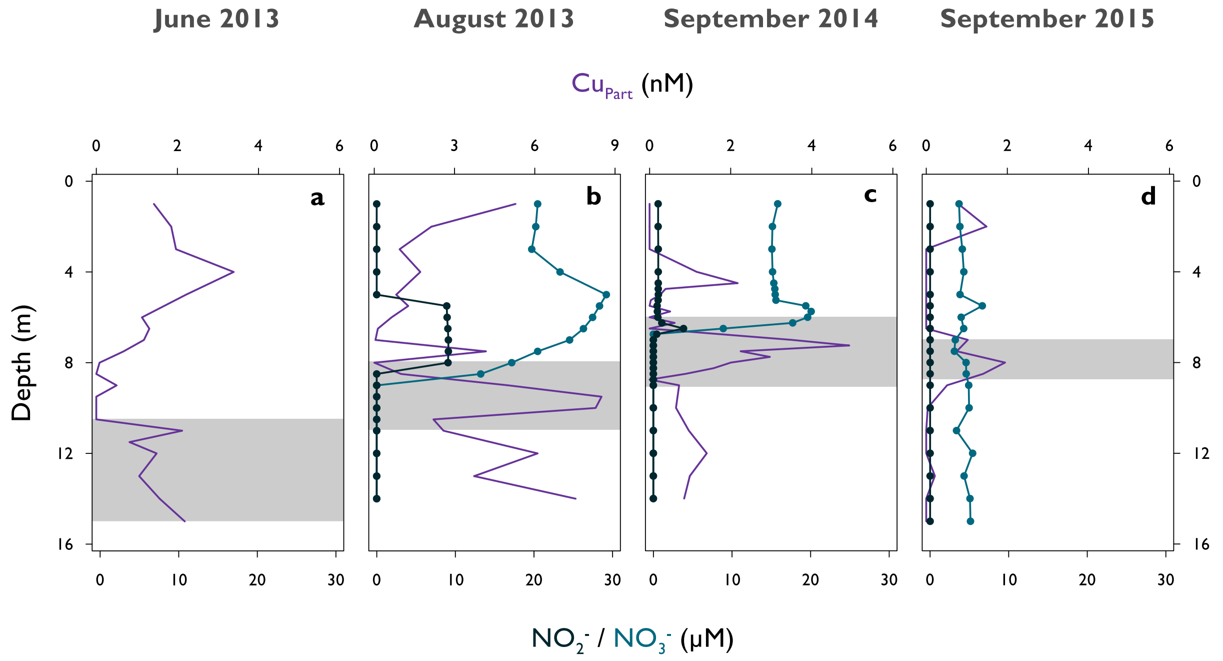

Figure S5. Ammonium and particulate copper concentrations in Rotsee. a, June 2013. **b**, August 2013. **c**, September 2014. **d**, September 2015. Ammonium (NH_4^+) in light turquoise, particulate copper (Cu_{Part}) in purple. Grey areas denote potential methane oxidation zones. Note the different x-axis scale for Cu_{Part} in August 2013 (**b**).

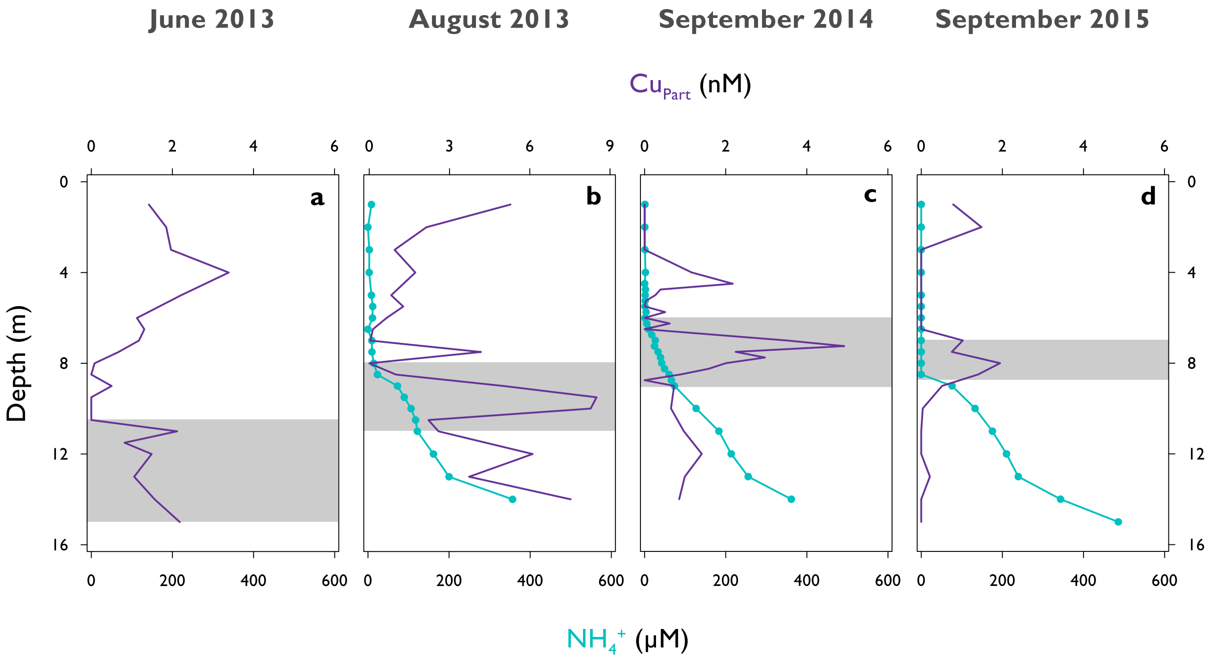

Figure S6. Depth profiles of particulate iron, manganese and copper in Rotsee. a, June 2013. **b**, August 2013. **c**, September 2014. **d**, September 2015. Particulate iron (Fe_{Part}) in black, particulate manganese (Mn_{Part}) in pink, particulate copper (Cu_{Part}) in purple. Grey areas denote potential methane oxidation zones. Missing data in Mn_{Part} profiles in the hypolimnion of June 2013 (**a**) are due to undiluted concentrations exceeding ICP-MS maximum detection limit. Note the different x-axis scale for Cu_{Part} in August 2013 (**b**).

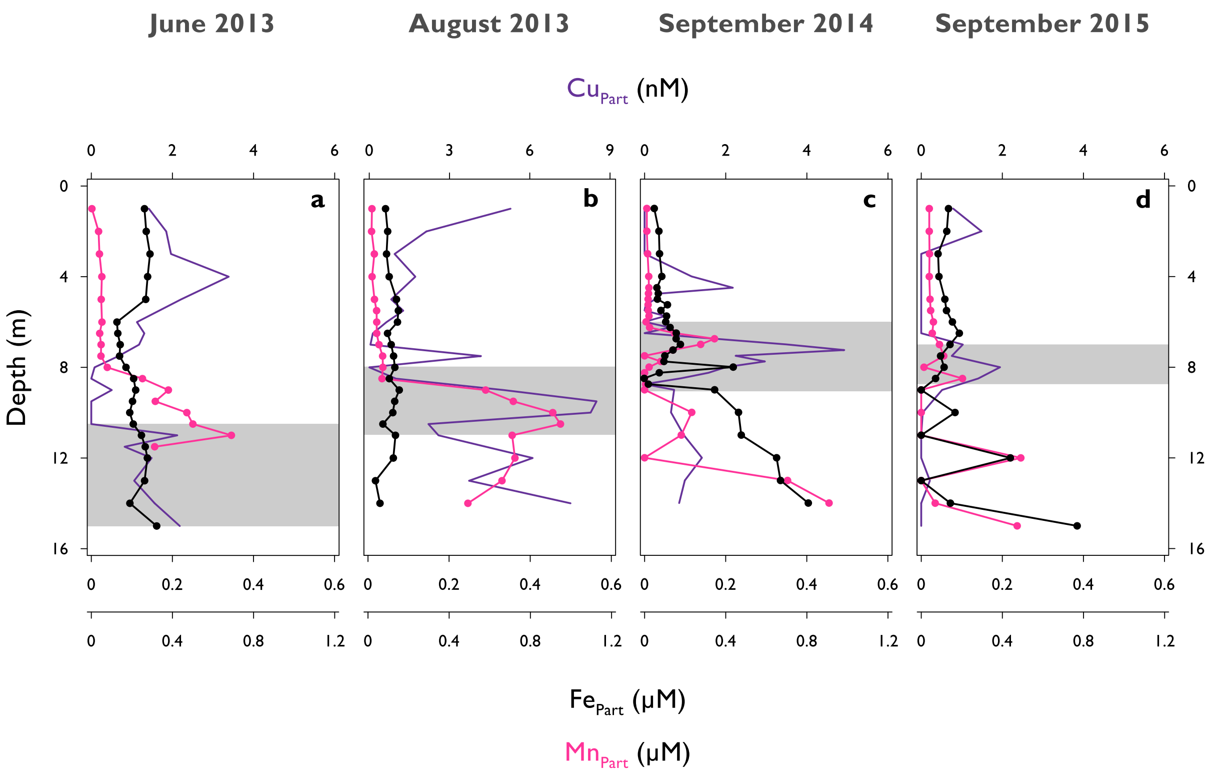

Figure S7. Total sulphide, sulphate, sulphur oxidizing bacteria and particulate copper profiles in Rotsee. a, June 2013. **b**, August 2013. **c**, September 2014. **d**, September 2015. Total sulphide (S_{Tot}) is shown in deep red, sulphate (SO_4^{2-}) in light red, particulate copper (Cu_{Part}) in purple. Relative abundance of sulphur oxidizing bacteria (SOB) are represented by the bar plot (see supplementary txt-file "16S rRNA sequences_SOB" for corresponding sequences). Grey areas denote potential methane oxidation zones. Note the different x-axis scale for Cu_{Part} in August 2013 (**b**).

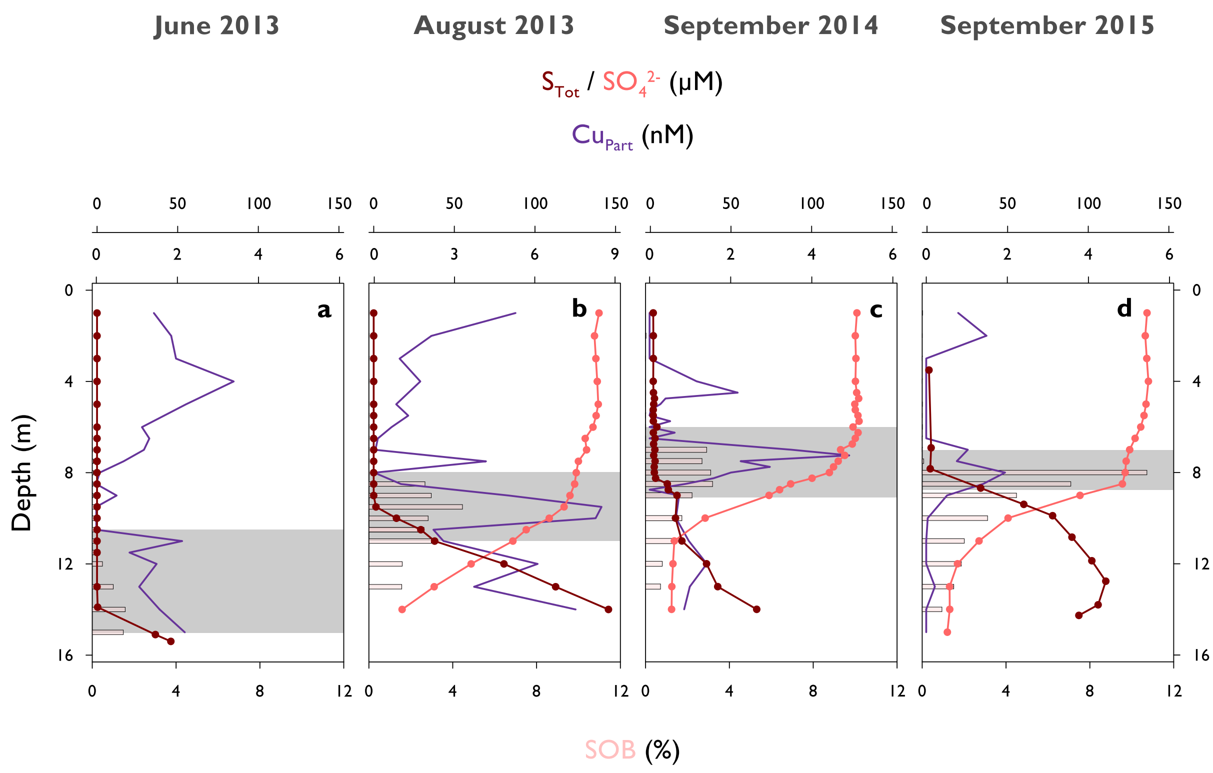

Figure S8. Diffusive Gradients in Thin film (DGT) samplers installation in Rotsee. The DGT parts consisted of a plastic piston, layered with a resin gel (red), a diffusive gel (deep grey) and a protective filter (light grey), and were covered with an open plastic cap. 3-4 DGT samplers were placed in a plastic stripe and attached to a rope, which was loosely connected to a floating buoy on top to place it in a straight condition.

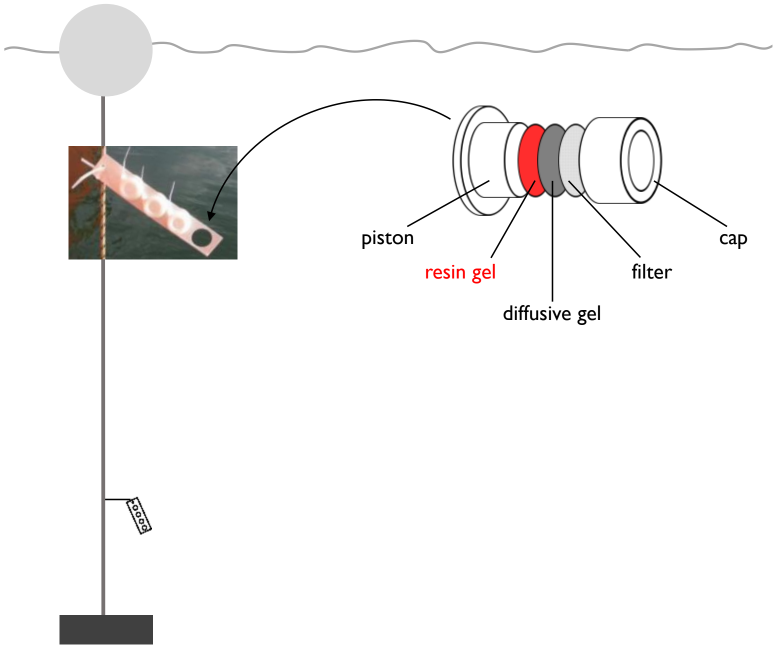

Table S1. Statistical tests describing bioavailable (Cu_{DGT}), dissolved (Cu_{Diss}), and particulate (Cu_{Part}) copper distributions within Rotsee.

Parameter	All	June 2013	August 2013	September 2014	September 2015
**Cu_{DGT}**					
Sample size (n)	284	54	73	81	75
Normality	$p < 0.001$	$p < 0.01$	$p < 0.001$	$p < 0.001$	$p < 0.001$
Kruskal-Wallis	$p < 0.001$	$p = 0.6$	$p < 0.001$	$p < 0.001$	$p < 0.001$
Mann-Whitney					
oxic - methane oxidation	$p < 0.001$		$p < 0.001$	$p < 0.001$	$p < 0.001$
oxic - anoxic	$p < 0.001$		$p < 0.001$	$p < 0.001$	$p < 0.001$
methane oxidation - anoxic	$p < 0.001$		$p < 0.05$	$p < 0.001$	$p < 0.001$
**Cu_{Diss}**					
Sample size (n)	260	60	60	83	57
Normality	$p < 0.001$	$p < 0.001$	$p < 0.001$	$p < 0.001$	$p < 0.001$
Kruskal-Wallis	$p < 0.001$	$p < 0.001$	$p < 0.001$	$p < 0.001$	$p < 0.001$
Mann-Whitney					
oxic - methane oxidation	$p < 0.001$	$p < 0.001$	$p < 0.001$	$p < 0.001$	$p < 0.001$
oxic - anoxic	$p < 0.001$		$p < 0.001$	$p < 0.001$	$p < 0.001$
methane oxidation - anoxic	$p < 0.001$		$p < 0.001$	$p < 0.001$	$p < 0.001$
**Cu_{Part}**					
Sample size (n)	88	21	20	28	19
Normality test	$p < 0.001$	$p = 0.21$	$p < 0.05$	$p < 0.001$	$p < 0.001$
Kruskal-Wallis / ANOVA	$p < 0.05$	$p = 0.66$	$p = 0.07$	$p = 0.07$	$p < 0.05$
Mann-Whitney / Tukey's HSD					
oxic - methane oxidation	$p < 0.01$				$p < 0.05$
oxic - anoxic	$p = 0.76$				$p = 0.84$
methane oxidation - anoxic	$p = 0.09$				$p < 0.01$

The water column was divided into three zones (oxic, methane oxidation, anoxic). Normality of the data was assessed by the Shapiro-Wilk test. Differences between zones were tested by one-way ANOVA followed by Tukey's HSD. When normality was not met, differences were tested by the Kruskal-wallis test followed by the Mann-Whitney pairwise test. p-values < 0.05 were considered significant.

Table S2. Relative abundances and phylogenetic affiliations of methane-oxidizing bacterial operational taxonomic units (OTUs) in Rotsee.

		OTU abundance (%)			
**OTU**	**Taxonomy**	**June 2013**	**August 2013**	**September 2014**	**September 2015**
OTU_129	α-MOB	0.018	0.394	1.144	5.542
OTU_56	γ-MOB	6.266	2.172	2.277	6.067
OTU_68	γ-MOB	8.713	0.753	2.941	2.993
OTU_141	γ-MOB	8.599	1.162	0.011	0.868
OTU_319	γ-MOB	0.024	0.006	1.175	0.050
OTU_433	γ-MOB	0.049	0.241	0.279	0.203
OTU_575	γ-MOB	1.440	0.846	0.081	0.364
OTU_614	γ-MOB	0.003	0.004	0.178	0.055
OTU_663	γ-MOB	0.002	0.143	0.048	0.074
OTU_821	γ-MOB	-	-	0.236	0.015
OTU_917	γ-MOB	-	0.002	0.072	0.020
OTU_931	γ-MOB	0.630	0.089	0.093	0.158
OTU_2276	γ-MOB	-	-	0.015	0.008
OTU_2426	γ-MOB	-	-	0.005	0.002
OTU_3248	γ-MOB	-	-	-	0.005
OTU_3421	γ-MOB	-	-	0.005	-
OTU_112	Methylacidiphilae	0.687	7.471	3.668	8.796
OTU_566	Methylacidiphilae	0.351	0.070	0.030	0.396
OTU_1479	Methylacidiphilae	0.013	-	0.012	0.016
OTU_1541	Methylacidiphilae	0.002	-	0.022	-
OTU_1686	Methylacidiphilae	0.005	0.003	0.020	0.006

γ-MOB: methane-oxidizing bacteria belonging to *Gammaproteobacteria*, α-MOB: methane-oxidizing bacteria belonging to *Alphaproteobacteria*. *Methylacidiphilae* group within *Verrucomicrobia*. After transforming absolute OTU reads to relative abundances, single OTUs are listed as sums of the water column for each campaign. See supplementary txt-file "16S rRNA sequences_MOB" for sequences corresponding to each OTU.

Table S3. Upward and downward fluxes of dissolved species in the water column of Rotsee.

Parameter		June 2013 (10.5-15 m)		August 2013 (8-11 m)		September 2014 (6-9 m)		September 2015 (7-8.7 m)	
O_2	(mmol m^{-2} d^{-1})	3.4	± 1%	31.3	± 3%	27.6	± 1%	42.9	± 1%
NO_2^-	(mmol m^{-2} d^{-1})	n.a.		1.6	± 0%	0.7	± 45%	0.0	± 0%
NO_3^-	(mmol m^{-2} d^{-1})	n.a.		1.5	± 31%	3.0	± 0%	0.0	± 0%
SO_4^{2-}	(mmol m^{-2} d^{-1})	n.a.		0.3	± 18%	0.9	± 10%	0.4	± 8%
Cu_{DGT}	(nmol m^{-2} d^{-1})	19	± 24%	30	± 21%	20	± 8%	40	± 9%
Cu_{Diss}	(nmol m^{-2} d^{-1})	195	± 8%	336	± 20%	828	± 25%	394	± 16%
CH_4	(mmol m^{-2} d^{-1})	-10.2	± 3%	-13.3	± 25%	-2.7	± 17%	-9.2	± 9%
Fe_{DGT}	(μmol m^{-2} d^{-1})	-13	± 20%	-13	± 25%	-47	± 5%	-12	± 11%
Fe_{Diss}	(μmol m^{-2} d^{-1})	-4.5	± 29%	-19	± 9%	-62	± 13%	-24	± 24%
Mn_{Diss}	(μmol m^{-2} d^{-1})	-52	± 21%	-22	± 49%	-136	± 5%	-87	± 5%
NH_4^+	(mmol m^{-2} d^{-1})	n.a.		-6.4	± 27%	-3.9	± 4%	-4.2	± 11%
S_{Tot}	(mmol m^{-2} d^{-1})	-2.6	± 1%	-3.1	± 5%	-1.1	± 16%	-1.8	± 14%

Oxygen: O_2, nitrite: NO_2^-, nitrate: NO_3^-, sulphate: SO_4^{2-}, bioavailable copper/iron: Cu_{DGT}/Fe_{DGT}, dissolved copper/iron/manganese: Cu_{Diss}/Fe_{Diss}/Mn_{Diss}, methane: CH_4, ammonium: NH_4^+, total sulphide: S_{Tot}. The depth ranges in brackets define the methane oxidation zones (grey bars in figures). Fluxes were calculated from the chemical concentration gradients determined by linear regression and the same vertical diffusion coefficient for all substances. n.a.: not analysed. Note the different units between the parameters.

Table S4. Primer pairs and amplification conditions for quantitative detection (qPCR) of 16S rRNA and MMO functional genes (*pmoA* and *mmoX*).

Gene	Primer	Sequence (5' to 3')	Reagents	Thermal profile & Quantification analysis	Efficiency
16S rRNA[1]	349f<br>806r	AGAGTTTGATCMTGGCTCAG<br>GGACTACCAGGGTATCTAAT	1 x master mix (LightCycler® 480 Probes Master, Roche)<br>0.9 µM primers (Microsynth)<br>0.3 µM TaqMan probe (Bac516F FAM; Microsynth)<br>2 µl DNA (1:100 diluted in AE)<br>10 µl final volume	Initial denaturation - 95 °C, 10 min<br>45 cycles - 95 °C, 40 sec; 53 °C, 40 sec; 72 °C, 1 min<br>Fluorescent reading after each cycle at 72 °C for 1 min<br>Absolute Quantification/2nd Derivative Maximum method	1.822
pmoA[2,3]	A189f<br>mb661r	GGNGACTGGGACTTCTGG<br>CCGGMGCAACGTCYTTACC	1 x master mix (LightCycler® 480 SYBR® Green I Master, Roche)<br>0.2 µM primers (Eurofins Genomics)<br>2 µl DNA (1:10 diluted in AE)<br>10 µl final volume	Initial denaturation - 95 °C, 10 min<br>10 touchdown cycles - 95 °C, 10 sec; 62-53 °C, 30 sec (-1 °C/cycle); 72 °C, 30 sec<br>30 cycles - 95 °C, 10 sec; 52 °C, 30 sec, 72 °C, 30 sec<br>Fluorescent reading after each cycle at 79 °C for 30 sec<br>Melting curve analysis after last cycle from 65-97 °C (0.11 °C/sec)<br>Absolute Quantification/2nd Derivative Maximum method	1.868
mmoX[4]	536f<br>898r	CGCTGTGGAAGGGCATGAAGCG<br>GCTCGACCTTGAACTTGGAGCC	1 x EvaGreen (Biotium)<br>1 x PCR buffer (Promega)<br>2 mM $MgCl_2$ (Promega)<br>0.2 mM dNTP (Qiagen)<br>0.25 mg ml^{-1} BSA (Sigma Aldrich)<br>0.27 µM primers (Microsynth)<br>0.025 U *Taq* polymerase (Go Taq G2 Flexi DNA Polymerase, Promega)<br>2 µl DNA (undiluted)<br>10 µl final volume	Initial denaturation - 95 °C, 5 min<br>38 cycles - 95 °C, 1 min; 63 °C, 1 min; 72 °C, 40 sec<br>Fluorescent reading after each cycle at 72 °C for 40 sec<br>Melting curve analysis after last cycle from 65-97 °C (0.11 °C/sec)<br>Fit Points method	2.018

Table S5. Taxonomic and physiological characteristics of axenic methane-oxidizing bacterial cultures.

Class	Species	Collection	*pmoA / mmoX*
α-MOB	*Methylocapsa aurea*	DSM 22158	y / n
	Methylocystis heyeri	DSM 16984	y / y
	Methylocystis hirsuta	R. Henneberger	y / y
	Methylocystis rosea	DSM 17261	y / n
	Methyloferula stellata	DSM 22108	n / y
	Methylosinus sporium	DSM 17706	y / y
	Methylosinus trichosporium	R. Henneberger	y / y
γ-MOB	*Methylococcus capsulatus*	R. Henneberger	y / y
	Methylomicrobium alcaliphilum	DSM 19304	y / n

α-MOB and γ-MOB are abbreviations for methane-oxidizing bacteria (MOB) belonging either to *Alpha-* or *Gammaproteobacteria*. Axenic cultures were either ordered from DSMZ (Deutsche Sammlung von Mikroorganismen und Zellkulturen) or received from R. Henneberger. y = organism contains respective functional gene, n = organism does not contain respective functional gene.

References

1. Takai, K. & Horikoshi, K. Rapid detection and quantification of members of the archaeal community by quantitative PCR using fluorogenic probes. *Appl. Environ. Microbiol.* **66,** 5066–5072 (2000).

2. Holmes, A. J., Costello, A. M., Lidstrom, M. E. & Murrell, J. C. Evidence that particulate methane monooxygenase and ammonia mooxygenase may be evolutionarily related. *FEMS Microbiol. Lett.* **132,** 203–208 (1995).

3. Costello, A. M. & Lidstrom, M. E. Molecular characterization of functional and phylogenetic genes from natural populations of methanotrophs in lake sediments. *Appl. Environ. Microbiol.* **65,** 5066–5074 (1999).

4. Fuse, H. *et al.* Oxidation of trichloroethylene and dimethyl sulfide by a marine Methylomicrobium strain containing soluble methane monooxygenase. *Biosci. Biotechnol. Biochem.* **62,** 1925–1931 (1998).
